# ‘It’s designed for someone who is not me’: A reflexive thematic analysis of the unmet healthcare support needs in UK autistic adults aged 65 years and over

**DOI:** 10.1177/13623613241291081

**Published:** 2024-10-29

**Authors:** Hassan Mansour, Amy Gillions, Jude Brown, Anoushka Pattenden, Susan Hartley, Sarifa Patel, Stephanie James, Martin Styles, Will Mandy, Joshua Stott, Elizabeth O’Nions

**Affiliations:** 1 University College London, UK; 2 National Autistic Society, UK; 3 Bradford Institute for Health Research, UK

**Keywords:** autism, health services, older adults, policy, qualitative research

## Abstract

Autistic people are more likely to experience physical and mental health difficulties across the lifespan, leading to an increased risk of premature mortality likely due to health disparities. At present, little is known about the healthcare experiences of autistic older adults. Semi-structured interviews were conducted with 19 autistic adults aged 65 years or over living in the United Kingdom. Reflexive thematic analysis produced these themes: (1) A lifetime of being misunderstood; (2) Autistic people falling between the gaps; (3) Processing autism diagnosis in later life; (4) Concerns about service pressures and scarce resources; (5) Lack of continuity of care, empathy and understanding; (6) Anxiety, alexithymia and sensory overload; and (7) Reduced social support and concerns for the future. Some of the challenges autistic older adults faced were like those experienced by younger or middle-aged autistic adults. However, they also reported additional difficulties related to ageing and lifelong experiences of social exclusion. These difficulties were made worse by increased service pressures. A comprehensive approach is needed, including enhanced staff training, personalised care, targeted support and policy changes. Services should also consult with autistic older adults so they can advocate for their care, shape policy and co-design services.

**Lay abstract:**

Autistic people often face both physical and mental health challenges throughout their lives, which can lead to a higher risk of premature death due to health inequalities. However, we know very little about the healthcare experiences of autistic older adults. In this study, we talked with 19 autistic adults aged 65 years or above living in the United Kingdom. We found these main themes: (1) A lifetime of being misunderstood; (2) Autistic people falling between the gaps; (3) Processing autism diagnosis in later life; (4) Concerns about service pressures and scarce resources; (5) Lack of continuity of care, empathy and understanding; (6) Anxiety, alexithymia and sensory overload; and (7) Reduced social support and concerns for the future. Themes show that autistic older adults face unique healthcare access challenges because services don’t consider their lifelong experiences of social exclusion. Participants also worried about age-related decline coupled with reduced social support, which makes it harder for them to get support. To address these challenges, healthcare services should provide more targeted support, make policies and funding consistent, and improve healthcare provision by providing staff training. Importantly, they must also speak with autistic older adults so they can advocate for their current and future care.

## Introduction

Autism is a neurodevelopmental condition characterised by social interaction and communication differences coupled with restricted, repetitive or stereotypic behaviour (American Psychiatric Association & Association, 2013). At least 1.1% of the English population meets diagnostic criteria for autism (Brugha et al., 2011), and 25%–32% of those individuals may also have a co-occurring intellectual disability (Rydzewska et al., 2019). While the number of adults receiving an autism diagnosis in England has increased over the past 20 years (Russell et al., 2022), most remain undiagnosed, with over 9 out of 10 autistic individuals over the age of 50 falling into this category (O’Nions et al., 2023). This is despite autistic adults experiencing significant functional, social and interpersonal challenges which persist across their lifespan (Atherton et al., 2022). In line with lived-experience research recommendations, identity-first language will be used throughout (Kenny et al., 2016).

‘Unjust and avoidable differences in healthcare access, quality, and outcomes’ have been found to impact autistic populations (Doherty et al., 2022; Scott & Rawal, 2018; Whitehead & Dahlgren, 1991). For example, autistic adults are five times more likely to experience poor general health and have a higher prevalence of physical health conditions such as diabetes, cardiovascular and gastrointestinal issues (Hand et al., 2020; Rydzewska et al., 2019). Up to 57% of autistic people meet diagnostic criteria for multiple co-occurring mental health conditions (Lever & Geurts, 2016). They also rely more on emergency care and experience a 6-year reduction in life expectancy (O’Nions et al., 2024; Vohra et al., 2016). With cause-specific mortality data indicating an increased risk of death across all ICD-10 categories except infections (Hirvikoski et al., 2016).

Autistic adults face patient, provider and system-level barriers when accessing healthcare services (Doherty et al., 2022; Walsh et al., 2020). Communication differences, sensory differences and alexithymia (difficulty in recognising or describing internal states) can make it difficult for autistic adults to seek support (Mason et al., 2021). Poor recognition and understanding of autism among providers often lead to a lack of adjustments or consideration for autistic patients’ needs, leaving them without appropriate support (Adams & Young, 2021; Doherty et al., 2020). System-level barriers include stigma around autism, lack of communication between services, and complex or non-existent referral pathways (Malik-Soni et al., 2022; Walsh et al., 2020). Reducing these barriers is vital, as negative experiences discourage autistic adults from seeking further support (Nicolaidis et al., 2015).

It is estimated that there are over 250,000 autistic adults aged 50 or older living in the United Kingdom (UK; O’Nions et al., 2023). However, services have a limited understanding of how to best support them (Mason et al., 2022). This lack of understanding is especially concerning for older people who tend to engage more with services and face age-related barriers such as physical and cognitive decline, which may interact with autism-specific barriers (Bishop-Fitzpatrick & Kind, 2017; Sonido et al., 2020). To our knowledge, no study has examined the access of older autistic adults to healthcare or how age-related needs interact with factors that disproportionately affect autistic individuals, such as reduced social support (Sonido et al., 2020; Wallace et al., 2016).

The current study aimed to explore the healthcare experiences of autistic older adults aged 65 years or over living in the UK, using in-depth semi-structured qualitative interviews. Given the inductive nature of reflexive thematic analysis, we had no specific hypothesis (Braun et al., 2023). Instead, we hoped that findings could help services improve experiences, access rates and treatment outcomes for autistic older adults.

## Method

### Ethics

The study received approval from UCL’s high-risk research ethics committee (22117/001).

### Setting

Participants were identified from an online survey on health and health literacy in autistic people aged 50 and above. The survey was advertised through third-sector organisations such as the National Autistic Society (NAS), Mencap, Autistica, Pathway Associates, Scottish Autism and the Autism Partnership Board. A study-specific social media profile was created to promote the survey among autism communities, including local Facebook groups, #AutisticElders, #ActuallyAutistic and #BlackAutistics. Participants who expressed an interest in the survey were screened against the following criteria: (1) having a diagnosis or self-identifying as autistic; (2) being at least 50 years old; and (3) accessing UK healthcare services. In the UK, almost everyone is registered with the National Healthcare Service (NHS), and access is free unless they actively seek out private care (Grosios et al., 2010).

After completing the survey, respondents indicated whether they would rather submit their answers anonymously or provide contact information to enter a draw for one of five £20 One4All gift vouchers. They were also given the option to take part in a follow-up qualitative interview. Responses were screened to identify and exclude potentially fraudulent participants (see Supplementary Section 1 for procedure details). Those who met the inclusion criteria and passed checks were then invited to participate in an interview. To ensure the inclusion of groups historically underrepresented in autism research, priority was given to participants aged 65 and over, identified as female or non-binary, and/or from ethnic minority backgrounds.

### Participants

Of the 188 individuals who completed the survey, 133 expressed an interest in participating in the qualitative interviews, with 27 being 65 years and over. Therefore, it was possible to select only participants aged 65 and over. Of these, 19 autistic adults aged 65+ were interviewed (see Table 1 for a summary of participant characteristics). We did not exclude participants who self-identified as autistic because there is evidence to suggest that there are barriers to accessing an autism diagnosis, particularly in adulthood, with most autistic older adults remaining undiagnosed (O’Nions et al., 2023).

**Table 1.** Summary of participant characteristics.

Characteristics	N (%)
**Age range (years)**	65-75
**Gender**	
Male	9 (47.4)
Female	7 (36.8)
Non-binary	2 (10.5)
Other (ungendered)	1 (5.3)
**Country of residence**	
England	16 (84.2)
Scotland	3 (15.8)
**Sexual orientation**	
Heterosexual	14 (73.7)
Homosexual	2 (10.5)
Asexual	1 (5.3)
Other	1 (5.3)
Prefer not to say	1 (5.3)
**Ethnicity**	
White British	17 (89.5)
Any other white	2 (10.5)
**Relationship status**	
Married	10 (52.6)
Single	4 (21.1)
Divorced	2 (10.5)
Widow	2 (10.5)
Separated	1 (5.3)
**Education level**	
Undergraduate	9 (47.4)
Postgraduate	4 (21.1)
Doctorate	3 (15.8)
School-age up to 18	1 (5.3)
School-age up to 16	1 (5.3)
Did not complete	1 (5.3)
**Autism diagnosis**	
Formal diagnosis	17 (89.5)
Self-identify	2 (10.5)
**Living situation**	
Alone	10 (52.6)
With spouse	8 (42.1)
With partner	1 (5.3)
**Employment**	
Retired	12 (63.2)
Employed (part-time)	3 (15.8)
Retired volunteer	2 (10.5)
Self-employed	2 (10.5)

### Procedure

Participants were invited for an interview and given at least 24 h to read the Participant Information Sheet. They were also asked if they required any adjustments, with some opting to preview the questions, while others preferred to split the interview into several parts. Interviews were conducted via telephone or online videoconference, recorded and transcribed securely. Each interview started with a general open-ended question about healthcare needs, followed by more specific questions about healthcare access challenges (see Supplementary Section 2 for the full interview schedule). Interview lengths varied from 45 min to 2 h, with H.M. completing all interviews. Upon completion, participants were offered a £15 One4All gift voucher.

### Analysis

Interview transcripts were analysed using reflexive thematic analysis via NVivo (Dhakal, 2022). Data analysis was conducted by H.M. using the six phases suggested by Braun and Clarke (2023): (1) Familiarisation with the data: as part of collection and interview; (2) Systematic data coding: two transcripts were double coded (by H.M. and A.G.) who discussed their codes. All interviews were coded line-by-line, with the coding framework iteratively revised; (3) Generating initial themes: concepts and ideas underpinning particular codes were identified around healthcare experiences; (4) Developing and reviewing themes: themes were visually mapped and consolidated; (5) Refining, defining and naming themes: central concepts or boundaries were considered for each theme; and (6) Writing the report: each theme and subtheme was populated with relevant extracts, and labelling of themes revised with input from J.S. and E.O.

### Reflexivity and ontology

H.M.’s interest in understanding the healthcare experiences of autistic older adults stems from his work within autism diagnostic services and his personal experiences of supporting family members with healthcare support needs to navigate services. As a non-autistic ‘outsider’ researcher, H.M. was mindful of the need for reflexivity (Braun & Clarke, 2023). By conducting a bracketing interview with A.G. and writing a reflexive journal, H.M. recognised his tendency to associate ageing and autism with increased healthcare difficulties. This led him to approach the interviews with a more open mindset, where he also enquired about strengths.

H.M. followed a critical realist ontological approach, which acknowledges the existence of one reality but recognises that different people or groups experience it differently (Scott, 2007). Semantic rather than latent-level coding was used to focus on the explicit meanings of the language used by participants rather than an interpretation of the meaning. This aligns with a contextualist epistemological stance, which considers the context in which language, knowledge and meaning exist (Madill et al., 2000). As such, H.M. chose a reflexive thematic analysis approach, which helped him consider how his values, experiences and clinical approaches might impact data analysis (Braun & Clarke, 2019; Braun et al., 2023).

### Community involvement statement

We consulted with a group of autistic older adults who formed part of an Experts by Experience Steering (EbE) group. They helped define the research aim and co-created all study materials. As such, H.M. reviewed the available literature and led an iterative consultation process to develop questions and identify potential topics to explore for each interview section (Clarke & Braun, 2013). The interview schedule was also piloted with an EbE member and further adjusted based on participant feedback.

## Results

**Figure 1.**
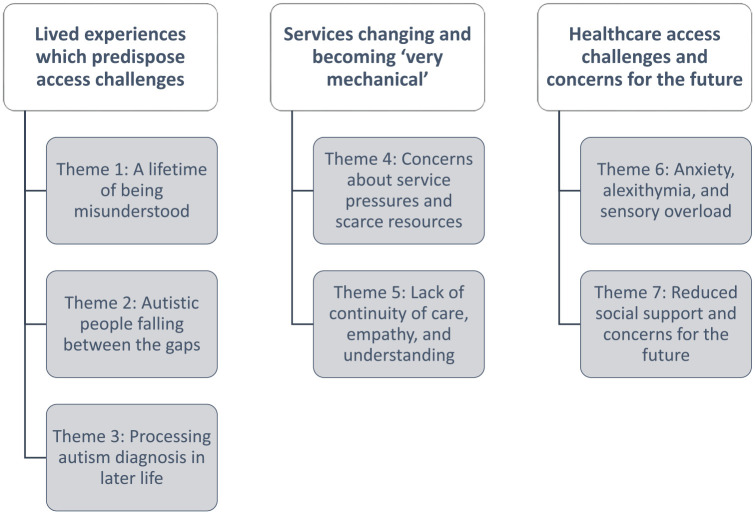
Organisation of headings and themes from reflexive thematic analysis.

We generated seven themes organised into three subheadings (see Figure 1).

### Lived experiences which predispose access challenges

#### Theme 1: A lifetime of being misunderstood

All participants’ accounts were of living most of their lives not knowing that they were autistic and trying to fit in. Growing up in an era when autism was little known provides insight into how a lifetime of being misunderstood has shaped their lives.

As children, many of them were repeatedly blamed for their differences: *‘I wasn’t diagnosed as a child which you should be these days. [. . .] Everybody thought I should be normal’ (P2)*; *‘[I] was frequently told I was a nuisance’ (P7)*. Attempts to pressure them to conform had often involved harsh criticism or abuse: *‘One of my teachers was disciplined for the extent of bullying to try to make me be like other children’ (P19)*. These past experiences of devaluation and denial of their autism impacted current interactions, including with healthcare staff: *‘I think I find it quite difficult to trust people and feel comfortable, you know? [. . .] Because I think I feel I won’t be taken seriously’ (P9)*.

As adults, most participants experienced anxiety and depression, which they linked to feeling different from others, and exhaustion from masking: ‘It just seemed like, although I was successful in my career, I was living this pretence, and it was so draining, so tiring, so stressful. And I think that’s where the depression came from, and still these feelings that you’re not the same as everybody else’. (P8)

One highlighted that anxiety was something that they had always lived with: *‘If you live in a world which makes you anxious all the time, it’s not a mental health problem. It’s just who you are’ (P7)*.

This anxiety was replicated when accessing services: *‘Your experience in the hospital is just exactly the same as your experience anywhere else, basically’ (P19)*.

#### Theme 2: Autistic people falling between the gaps

Many were left to manage their mental health difficulties on their own, with services often discharging participants.

This was due to poor recognition or understanding of autism and inadequate service provision: *‘The local health service authority has dismissed me from the mental health department three, maybe four times without explanation’ (P14)*.

When accessing mental health services, participants’ autism had often remained undetected or misdiagnosed: *‘He [a psychiatrist] didn’t understand me at all and misdiagnosed me with a personality disorder’ (P3)*.

There was also a sense that many autistic people without a co-occurring learning disability were overlooked: *‘In some parts of the country, they put you [autistic people] under mental health but in our part, you’re put under learning disability but if you need a counsellor or anything, you’re not allowed to use their resources if you haven’t got the learning disability as well’. (P6)*

Participants also highlighted the lack of health or social care services available: ‘I’ve had no help. I still don’t get any help. The only help I get is the stuff that I get from people I found on Zoom, from autistic groups. That’s the only help I get. And knowing that I’m not alone and there are other people out there’. (P2)

There were also difficulties getting an assessment: ‘My wife observed that I have similar traits [to my autistic son], and so I went to the GP and asked for a referral. It took six years and a change in the law, because they said that [only] if you have learning difficulties, [. . .] you could be assessed’. (P12)

One participant reported how inconsistent policy has potentially led to a reluctance to disclose his diagnosis:

*‘I have chosen, at this point, probably more out of procrastination than deliberately, not to tell my GP about my diagnosis. Because in accordance with Scottish law, and I’m not sure about this but it’s what I’ve gathered from reading, autism is, per se, a sectionable diagnosis’. (P5)*

#### Theme 3: Processing autism diagnosis in later life

Most participants found autism to be a helpful explanation for their differences, and something that had led them to view themselves in a more compassionate light: *‘I’ve had 30,000 questions as to why my life has been hell and the word autism came into my head. [. . .] it suddenly answered those 30,000 questions’ (P2)*. However, some experienced resistance to the idea that they are autistic: *‘Because my diagnosis was so late, there’s some part of me that still thinks I can make everything all right and just be normal’ (P9)*. Participants also reported unmet needs around the impact of being diagnosed:

*‘[the autism diagnosis] made me feel like I had to look at my life. I had to review my whole life through the lens of autism. And, I don’t know, you’re very confused. [. . .] You’re just left with all those feelings that you don’t know what to do with them’. (P16)*

It was therefore concerning that participants had needed to process the diagnosis and what that meant for them with little or no support.

Participants were also acutely aware of the stigma surrounding autism: *‘However well you know someone, it always affects, it seems to affect how they behave towards you. [. . .] It’s just as though they think you’re somehow not quite all there’ (P9)*.

Some participants had not disclosed their diagnosis even to those closest to them: *‘I haven’t told my mother. I haven’t told my sister. I haven’t told the people at work. And the reason for that is, it’s because I don’t feel safe to do that’ (P16).*

Having a record of autism on their medical records was also concerning for some: *‘At the time I had an eating disorder, anorexia, on my notes. And that’s always a stigma, people always look at you as if you’re a piece of rubbish. Of course, they now look at me as double rubbish because I’m autistic and I’ve got an eating disorder’. (P3)*

### Services changing and becoming ‘very mechanical’

#### Theme 4: Concerns about service pressures and scarce resources

All participants expressed concerns about the increased pressure on services and reduced resources. This led to fears about being a burden: *‘If it turns out that whatever it was [I went in for] isn’t significant, I feel absolutely terrible that I’ve wasted time and money, and I don’t want to do that’ (P19)*.

As a result, many were reluctant to access services: *‘I don’t go to the doctor unless I think it’s really urgent’ (P4)*.

When they did access services, most expected sub-optimal care: *‘I know my practice must be so overworked in terms of the GP-to-client ratio. They just don’t have the time or resource to do what they might have done in the past’ (P17)*.

There was also a sense that older adults were not the priority: *‘Sometimes it feels like if you’re above a certain age, your health doesn’t, it’s not that it doesn’t matter but it’s not as important as if you’re young’ (P16)*.

Participants spoke about preferring face-to-face over telephone consultations, given that in-person interactions offer cues that can help to determine underlying meanings, and reduce anxiety through co-regulation: *‘If I’m talking to somebody and they don’t get what I’m trying to say, I can literally go from nought to 20, and panic. [. . .] Whereas if I was seeing them face-to-face, they could just say a few calming things’ (P3)*.

This was concerning as face-to-face appointments had become less accessible since the pandemic. However, participants also stated that it was helpful for the initial contact to be via text or email: *‘But now I can put a request in, that I can think about, as an email. And somebody will get back to me, as an email [. . .]. So, in a sense, that builds in the pauses. That is more helpful for me’. (P13)*

But there were also queries about who might be left behind: *‘There are people who are older or people who’ve got conditions which means they can’t interact with these types of things [online services], so where do they go?’ (P15)*.

Increasing waitlists for treatment that could improve quality of life was also a concern, with participants expressing frustration about a lack of communication from services: *‘At one stage, months ago, I said, how long is the waiting list now? Because you used to say two years. And they simply said, oh, we’ve stopped telling people’ (P14)*.

This led to increased anxiety: *‘I’d much rather know whether it’s two months or two years because it’s not knowing. And I find not knowing things really, really hard’ (P9)*.

The sense of scarcity around resources also exacerbated participants’ anxiety when communicating with their GP: *‘[Getting a call] is obviously very difficult for me because I then am in anxiety all day. [. . .] Is my phone working? Is the internet working? [. . .] And then when she’s speaking to me, have I remembered everything I wanted to say? Did I say it in the right order?’. (P11)*

#### Theme 5: Lack of continuity of care, empathy and understanding

Participants noticed that high turnover and service restructuring meant that it was difficult to form relationships with staff.

The resulting lack of residual knowledge reduced the likelihood of reasonable adjustments: *‘I’ve had to go back three times [for blood tests], and they’ve said, oh, we haven’t got an appointment for two weeks and I’m absolutely terrified for two weeks, and I mean terrified, meltdowns, everything [. . .] my GP that I had before wouldn’t have done that. [. . .] he would put me in somewhere the next day’. (P6)*

For this reason most participants reported that seeing someone who knows them reduces their anxiety, particularly when seeking support for complex health issues.

Continuity of care was also important as knowledge gaps were common: *‘But I think there’s very little awareness in healthcare about autistic needs. [. . .] I’ve worked in healthcare since 1975 and I’ve had no training about autistic needs [. . .] And I know GPs, what do they get? About an hour?’ (P16)*.

Misunderstandings based on outdated ideas of autism were consistently reported: *‘. . . people [including healthcare professionals] don’t understand the breadth of autism [. . .] Like the way that you can be really normal seeming in their eyes and then really are very distressed about certain things’ (P10)*.

This would often lead participants to feel like they were not believed or viewed negatively when disclosing their autism.

For these reasons, long-serving, empathetic staff were highly valued: ‘My GP retired. Now it’s absolutely tragic [. . .] she was the first person that ever made us feel positive, or less negative about our eating disorder. [. . .] She said, you do know that an eating disorder’s not your fault, don’t you?’. (P3)

Participants identified empathic professionals by their receptivity and willingness to offer reassurance: *‘If I say, I’m autistic and I also have anxiety, they say okay, anytime that you feel you want to stop. [. . .] The ones that are genuine, they will, whatever they’re doing, they’ll say, is this, okay? Is that okay? I’m just going to hold your hand, they will explain to you, I’m just going to read your pulse here’. (P11)*

### Healthcare access challenges and concerns for the future

#### Theme 6: Anxiety, alexithymia and sensory overload

Participants were acutely aware that accessing healthcare inherently exposes them to novel and uncertain situations: *‘So, you go to the GP, or if you go to the hospital, what’s going to happen? I don’t know what they’re going to do to me. [. . .] It’s new surroundings, new people. All those things add to your overall overload and anxiety’. (P8)*

Anxiety represented a challenge because it made it harder to communicate: *‘If I’m talking to a doctor I’ve got this mental list of things I need to say [. . .] And I often forget, sometimes, even the most important things, I forget to say’ (P9)*.

Some participants used strategies such as rehearsing what they might say in advance, though this was not always effective: *‘When you’re all revved up to say what you want to say, and you walk in [the doctor’s room], and they go I’m just looking at your notes. So your impetus has gone. This is wrong. It shouldn’t have happened, because it wasn’t in your rehearsed pattern’. (P7)*

Some participants reported that they struggle to notice and communicate emotions (‘alexithymia’): *‘That’s probably a reason why they [the psychiatrist] couldn’t sort me out because I couldn’t tell them how I was really feeling. Because you say to me, how do you feel and I’ll say fine’ (P2)*.

Some also struggled to recognise and communicate bodily states (‘interoception’): *‘I’ve been walking on a sprained ankle for ten years. I don’t notice that stuff, basically. It’s not that I can’t feel it, it’s just the brain doesn’t pick up the signal’ (P19)*.

Differences in non-verbal communication also made seeking support difficult: *‘You can see they’re [non-autistic people] in pain, but you can’t see I’m in pain because our faces could be the same for happy things or sad things [. . .] So they don’t get how bad you are’ (P6)*.

Sensory overload also made accessing services and undergoing certain procedures a challenge: *‘MRI scans are very difficult actually because they’re so loud. But it’s the MRI scans that have confirmed what my diagnosis is [arthritis], so it’s a payoff isn’t it?’ (P13)*.

All the above feed into each other and combine: ‘[Upon entering the hospital corridor] you start spinning around, making noises. Going into meltdown because of all the people there and the noise and smell. And then if I ask directions, they give you three or four ways to go up the lift and God knows what, and I can only remember the first one. And then I can’t go in a lift on my own and it was upstairs. You had to go in the lift. I couldn’t have gone up all the stairs. [. . .] So I just ended up not being able to drop it [urine sample] off’. (P6)

#### Theme 7: Reduced social support and concerns for the future

Some participants reported increased confidence in requesting accommodations as they got older: *‘I found, as I aged, that it’s become more and more exhausting. [. . .] I have learnt to do things like when I know that something will be too much, to just say no, I’m sorry. I can’t do that’ (P7)*.

However, participants expressed concerns about who would support them in the future: *‘We, mostly, have fairly tortured relationships with our families who regard us as a burden. We, usually, have quite small circles of friends. A lot of us are married, but a lot of us aren’t. And a lot of marriages break down and a lot of us are non-binary which adds an extra layer to it. And I think it is extremely worrying contemplating where autistic people are going to be when incapacitated’. (P19)*

These concerns were especially relevant to the impact of age-related decline: *‘When I’m more elderly and less able, mentally and physically, now my life is in somebody else’s hands to a degree. Now, if that person [. . .] they are not autistic aware or haven’t had training [. . .] I’m just going to be their worst nightmare, aren’t I? [. . .] If you try to make me do something I don’t want to do, I might not have the capacity to explain to somebody, I don’t like this because of the noise, or, I don’t like this, stop touching me. [. . .] If I haven’t got the capacity to do that, well, that’s me locked into hell’. (P8)*

Participants also highlighted that social isolation was something that health providers should consider: *‘Older autistic people might be more isolated than older typical people. And so therefore it’s kind of important [for healthcare staff] to see the whole person and not just the medical condition [. . .] there may not be the same support that a typical person would have in terms of family’. (P1)*

##### Recommendations for service development

Participants offered suggestions for improvements that could facilitate engagement and reduce anxiety (see Table 2). These recommendations were co-constructed through reflexive thematic analysis, with a table of quotes being found in Supplementary Section 3.

**Table 2.** Summary of healthcare recommendations and quotes made by participants.

**Comprehensive staff training:** Develop and implement training programmes that equip healthcare professionals to better understand and support autistic needs or experiences, including communication or sensory differences.	‘I know that GP practices reception staff are meant to have some form of training in autism. But I mean, I think I’d like to know how far that goes and how far they can actually apply that in the situation’. (P1)
**Consistent policy:** Develop consistent policies that prioritise the needs of autistic individuals at a national level. This might help address some of the systemic barriers to access that many autistic people experience across the UK, especially when it comes to accessing services or funding.	‘In some parts of the country, they put you under mental health but in our part, you’re put under learning disability but if you need a counsellor or anything, you’re not allowed to use their resources if you haven’t got the learning disability as well’. (P6)
**Proactive care:** Establish specialised, targeted support services for autistic older adults. These may include regular check-ins, updates about waitlist positions, autism-specific support groups and tailored support to combat negative experiences with services and social isolation.	‘If just someone [from the GP surgery] could [make contact by phone]. Not all the time but just now and again, check in and it’s, how are you doing? Have you got any problems? Is there anything, we need to send you to help you? That sort of thing would be brilliant because then you don’t feel as though you’re being a nuisance’. (P9)
**Sensory-friendly practice:** Environmental factors such as temperature, lighting, noise and overcrowding should be considered. Similarly, staff should be mindful of differences in touch, smell, noise, light, and taste or pill dysphagia.	‘I suffer from sensory overload, I would like that to be sorted out. A facility where I can be kept quiet, no bright lights and people rushing around in front of me. Ideally, to be put into a single room [. . .] shielded from the rest of the noise’. (P2)
**Empathy and reassurance:** Provide clear reassurance, empathy and explanations to help reduce the power imbalance many older adults with autism might experience throughout their lifetime. This could be due to them being disbelieved and stigmatised by family, peers and figures of authority such as teachers, employers and especially healthcare professionals.	‘It’s a question of just getting to know that person, even right at the start of the conversation just call them by their name. Maybe a couple of comments about a past experience, just to signpost them that you know this person, and maybe a couple of reassuring comments that, when you do come in, you’ll be seeing this person, and that’s going to happen’. (P3)
**Communication and processing:** Be mindful of communication, processing or learning differences. Adjust according to visual or auditory learning style, allow extra processing time, check understanding and adapt questioning styles (e.g. use close-ended questions if necessary).	‘I think just needing a little bit more time, and probably making sure that what they have said has been properly understood. Because the language thing can be a big thing. [. . .] I take it literally, and then discover much later that that’s not what they meant’. (P7)
**Continuity of care:** Assign the same healthcare professional so there is continuity of care. If this is not possible, read the notes and ask the autistic person if they have any needs or adjustments. Also, check in with different healthcare services or staff to ensure more seamless care.	‘As soon as they see [. . .] on your records, on your piece of paper, a little autism sign or something. [. . .] That they go, oh yes, click. Oh right. Now I need not just to refer to the last GP’s notes [. . .] I need, for this guy, to see the same person every time. And this never happens’. (P14)

## Discussion

The current study is the first to our knowledge to investigate the healthcare experience of UK autistic adults aged 65 years and above (Sonido et al., 2020). Participants lived much of their lives with autism not widely discussed and narrowly defined. Using reflexive thematic analysis, we identified the following themes: (1) A lifetime of being misunderstood; (2) Autistic people falling between the gaps; (3) Processing autism diagnosis in later life; (4) Concerns about service pressures and scarce resources; (5) Lack of continuity of care, empathy and understanding; (6) Anxiety, alexithymia and sensory overload; and (7) Reduced social support and concerns for the future. Findings highlight how these factors intersect and predispose autistic adults to poorer health and greater difficulty accessing care. Participants also identified significant gaps in current service delivery and suggested possible ways to address them.

### Comparison with other studies

#### Lived experiences which predispose access challenges

Several of the identified themes overlap with previous research on younger and middle-aged autistic adults. These include autistic people falling between the gaps, lack of continuity of care and challenges with anxiety, alexithymia and sensory overload (Mason et al., 2021; Walsh et al., 2020). However, our study is unique in highlighting the long-term impact of growing up and living most of one’s adult life without knowing about autism and being misunderstood. As reported elsewhere, many participants had been targeted and blamed for their differences (Pearson et al., 2023), experiencing social exclusion and financial instability (Davies, Romualdez, et al., 2024). This coupled with a societal lack of autism awareness left many feeling vulnerable, different and anxious since childhood.

Participants reported that their mental health needs remained unmet as they were unable to access support due to inconsistencies in policies and service provision. This is in line with other studies which suggest that mental health services are often ill-equipped to cater to the needs of autistic individuals as they are under-resourced and staff can fail to make adjustments (Brede et al., 2022). As a result, participants had to manage alone, with services discharging or misdiagnosing them, including applying stigmatising labels like borderline personality disorder (Fusar-Poli et al., 2022). This, coupled with a lifetime of being misunderstood even by those in authority, meant that accessing support was particularly anxiety-provoking. For this reason, many valued when professionals showed empathy and explicit reassurance as this helped reduce the sense of power imbalance.

As reported elsewhere, many participants embraced being autistic as a positive aspect of their identity (Davies, Cooper, et al., 2024; Hickey et al., 2018). However, for some, the diagnosis came with mixed emotions, especially if they had internalised societal stigma about autism. As such, there was an urgent need for post-diagnostic support, which has consistently been found to be lacking (Crane et al., 2018). This was also apparent among the older adults we interviewed, with autism support groups being non-existent in certain regions. For some, joining an online group was the only way to connect with other autistic people. However, many were looking for local in-person groups, particularly where they could meet other autistic older adults.

#### Services changing and becoming ‘very mechanical’

Similar to research on younger and middle-aged autistic adults; system and service-level barriers emerged as important factors that influence overall healthcare experiences (Mason et al., 2021; Walsh et al., 2020). However, participants also expressed concerns about the increased pressure on healthcare services and many were worried about being a burden or wasting resources. While this may not be unique to autistic older adults, there was a sense that healthcare services and society, in general, did not prioritise the needs of older adults. This, coupled with the fact that novel situations were particularly stressful, meant that many participants would delay accessing support unless it was urgent.

Changes to service design that facilitated communication with the GP through email or especially text messages were described as helpful (Doherty et al., 2022). However, other changes, such as longer wait times, reduced updates, disjointed care, limited face-to-face appointments and complicated care pathways, were a concern. There was a general perception that healthcare is becoming more ‘mechanical’ and less empathetic. Like findings from autistic younger adults, the presence of a consistent patient-provider relationship was crucial in building trust, familiarity and personalised care (Mazurek et al., 2023). This need for consistency may be particularly important in later life, where there might be more complex healthcare needs.

#### Access challenges and concerns for the future

Sensory differences, anxiety and alexithymia were all listed as barriers to accessing healthcare services. These findings are consistent with previous studies in younger or middle-aged adults (Calleja et al., 2020; Walsh et al., 2020). However, participants also described how the anxiety of entering a novel situation can worsen social communication and cognitive processing difficulties. To manage, some participants use compensatory strategies such as practising what they might say in advance. However, this can increase cognitive load and make it more difficult to remember what was discussed (Livingston & Happé, 2017). All the above, coupled with having more chronic health conditions, can make it more difficult to access services.

To our knowledge, this is the first study to report autistic older adults’ concerns about the future. Participants highlighted the need for services to see ‘the whole person and not just the medical condition’, particularly for autistic older adults who have reduced social support. There were also significant concerns over the impact of age-related cognitive and physical decline, which could render them less capable of self-advocacy or utilising coping mechanisms they have developed. Studies have shown that smaller social support networks can lead to adverse outcomes in later life (Golden et al., 2009; Sakurai et al., 2019), and this could be especially relevant for autistic older adults who may experience social isolation.

#### Policy and practice recommendations

Consistent with other studies, participants emphasised the need for improved training so that staff can develop a better understanding of the heterogeneity of autism and the impact that small adjustments can have on improving healthcare experiences (Nicolaidis et al., 2015; Pilling et al., 2012). Participants also identified a need to involve autistic older adults and their families in service design plus the creation of consistent policies which mitigate gaps in service delivery across different regions. This is particularly important in the context of the limited health and social care services which are currently available for autistic adults across the UK (Crane et al., 2018). For the above reasons, participants identified a need for healthcare services to assess the support structures available to autistic older adults, who may be more isolated than their neurotypical peers (Wallace et al., 2016).

Recommendations included regular check-ins by general practice or social care staff. A need for support to coordinate complex care pathways was also identified. During direct contact with patients, healthcare staff should adjust their communication and language to align with the communication differences and preferences of the autistic person. This can include using unambiguous words with no metaphors, visual or written cues to explain abstract concepts, and closed-ended questions if someone is struggling to answer. Similarly, all participants highlighted the importance of having additional processing time to help reduce anxiety and improve comprehension. Moreover, familiarity and knowing what to expect before, during, and after the consultation were deemed essential to high-quality care.

### Implications

The present findings highlight significant unmet needs of autistic older adults, which may also impact other groups who have difficulties with communication, anxiety and processing speed. Autistic older adults experience substantial barriers to care, meaning that treatable problems may go unaddressed. At least some of these challenges can be overcome by implementing simple approaches, such as training healthcare staff to communicate inclusively, checking understanding, allowing enough processing time and making appropriate adjustments according to individual preferences or needs.

In line with the Lancet Commission’s recommendation for ‘a novel, modified stepped care and personal health model of intervention and assessment’ (Lord et al., 2022), the present findings highlight a need for specialist support. There is currently little guidance on accessing post-diagnostic support, with most autism-specific services focusing on children (Huang et al., 2020). In addition, primary healthcare services are often ill-equipped to deal with autism-specific concerns, while specialist services exclude many autistic adults for not meeting symptom or functional impairment thresholds (Brice et al., 2021). As a result, autistic adults are often left to deal with difficulties on their own (Crane et al., 2018), resulting in debility, poorer quality of life and avoidable deaths (White et al., 2023).

### Strengths and limitations

A strength of the present study was that we obtained detailed information about the lived experiences of autistic people aged 65 years and over, including those from typically underrepresented genders and sexual orientations that have not been included in autism research. However, the representation of ethnic minority backgrounds was limited. Moreover, by using an online survey to recruit participants, we may have introduced selection bias, potentially leading to the recruitment of participants interested in the topic and more engaged with healthcare services (Delgado-Rodriguez & Llorca, 2004). This is somewhat suggested in our sample characteristics, with many participants having an autism diagnosis in later life.

Online recruitment might also have excluded people who lack Internet access, have more significant challenges with language comprehension and those with more substantial health problems. Therefore, our findings reveal some but not all unmet health support needs likely experienced by autistic older adults. For instance, research has shown that people with learning disabilities, many of whom are also autistic, face other issues like overshadowing of physical health concerns by mental health conditions or ‘behaviours that challenge’, and overmedication with psychotropic drugs (Branford et al., 2018). Similarly, our findings are specific to the UK where healthcare is free to access at the point of contact through the NHS. As such, it would be important for future studies to examine whether findings are generalisable to broader autistic populations and countries where universal healthcare is unavailable or better funded.

## Conclusion

There are general challenges to being an NHS service user, especially in older adulthood and the context of reduced economic resources. However, these challenges are especially difficult for autistic people when considering that healthcare services are not designed with them in mind. As such, autistic people have had to develop coping strategies to navigate individual, service and system-level barriers. Moreover, as participants get older, they experience age-related cognitive and physical decline, which makes it even more difficult to use their coping strategies to navigate services. This is concerning, especially as many have reduced social support. As such, healthcare services need to provide more autism-friendly care, which includes targeted support, environmental adjustments, consistent policies and enhanced training for staff. More also needs to be done to include autistic older adults and their families in research so they can advocate for their care, shape policy and co-design services.

## Supplemental Material

sj-docx-1-aut-10.1177_13623613241291081 – Supplemental material for ‘It’s designed for someone who is not me’: A reflexive thematic analysis of the unmet healthcare support needs in UK autistic adults aged 65 years and over Supplemental material, sj-docx-1-aut-10.1177_13623613241291081 for ‘It’s designed for someone who is not me’: A reflexive thematic analysis of the unmet healthcare support needs in UK autistic adults aged 65 years and over by Hassan Mansour, Amy Gillions, Jude Brown, Anoushka Pattenden, Susan Hartley, Sarifa Patel, Stephanie James, Martin Styles, Will Mandy, Joshua Stott and Elizabeth O’Nions in Autism
