# Supplementary material for: ‘It’s designed for someone who is not me’: A reflexive thematic analysis of the unmet healthcare support needs in UK autistic adults aged 65 years and over
Source: Autism. 2024 Oct 29;29(3):754–65. doi: 10.1177/13623613241291081 (PMC11894832; doi:10.1177/13623613241291081)
Supplement: sj-docx-1-aut-10.1177_13623613241291081 – Supplemental material for ‘It’s designed for someone who is not me’: A reflexive thematic analysis of the unmet healthcare support needs in UK autistic adults aged 65 years and over [file sj-docx-1-aut-10.1177_13623613241291081.docx]

**Supplementary Section 1. Procedure to identify and exclude potentially fraudulent responses:**

To identify and control for possibly fraudulent responses, we contacted Qualtrics and asked them to enable any safety features that could screen or check for bots. All participants had to complete a captcha test before starting each survey. We also asked to access survey response data associated with time and location. This enabled us to check how long each person took to complete the survey and whether the same IP address was used for multiple survey responses. Similarly, we checked all questionnaires to see if participants selected the same answer throughout or missed large chunks of questions. Finally, we included several open-ended questions about healthcare needs and experiences. This was perhaps the most accurate way to check for fraudulent responses, as the answer would be overly vague or generic, especially compared to other more detailed responses.

**Supplementary Section 2. Interview schedule used for the study:**

**Healthcare needs:**

- What are your current healthcare needs?
  - Do you have any physical or mental health difficulties?
    - If yes, then how easy was it to access support?
  - Do you have any current concerns about your healthcare needs?
- What have been your healthcare needs been in the past?
  - Have you received a diagnosis for a condition which you felt was inaccurate?

**Access to services:**

- How often do you access healthcare services?
  - What is it like contacting your GP?
  - Have you been to hospital in the last two years?
    - If yes, what was this experience like?
- How do you experience services which offer healthcare support?
  - How do you feel you are treated?
  - Do you feel like they listen or understand you?
  - Do you feel respected?
- How have your healthcare needs changed, particularly as you have gotten older?
  - How do you think your experience of services or accessing services compares to when you were younger?

**Barriers and facilitators:**

- Have you experienced any difficulties when accessing or engaging with services?
  - What are the barriers which prevent you from accessing healthcare services?
  - What helps when trying to access or engage with healthcare services?
- How do other aspects of your identity/life influence your healthcare needs and access to services?
  - Ethnicity, religion, gender, sexuality, socioeconomic status, and spirituality
  - Wider social network including family, friends, and support groups
- How have services tried to adapt to meet your needs?
  - What helps facilitate access to healthcare services, particularly in older age?
    - Prompts: communication/literacy, explaining things in different ways
- What strengths and resources do you think might come with ageing?

Summing up:

- How could healthcare services be improved for older autistic people?

- Is there anything important that we have not discussed or asked?

**Supplementary Section 3. Healthcare recommendations made by autistic older adults:**

| 1 | Comprehensive  staff training | “People [including healthcare professionals] don’t understand the breadth of autism […] the way that you can be really normal seeming in their eyes and then are very distressed about certain things” (P10)  “I’ve worked in healthcare since 1975 and I’ve had no training about autistic needs or no training about looking after patients that are autistic. And I know GPs, what do they get? About an hour?” (P16)  “I don’t feel people understand learning disabilities, and I don’t think people understand autism, and I don’t think people understand trauma and the impact of it on people with learning disabilities.” (P20) |
| --- | --- | --- |
| 2 | **Consistent policy** | “I have chosen, at this point, probably more out of procrastination than deliberately, not to tell my GP about my diagnosis. Because in accordance with Scottish law, and I’m not sure about this but it’s what I’ve gathered from reading, autism is, per se, a sectionable diagnosis.” (P5)  “My youngest son was diagnosed with Asperger’s when he was 12. My wife observed that I have similar traits, and so I went to the GP and asked for a referral. It took six years and a change in the law, because they said that if you have learning difficulties, then you could be assessed.” (P12)  “The 2010 Equalities Acts that’s just transformed my life completely. […] They can’t openly discriminate against you. You can just tell them, and that takes a lot of the problem out of it.” (P19) |
| 3 | **Proactive care** | “Just general communication about what’s happening. Like, maybe, just sending a quick email […] saying, oh, you’re now here on the waitlist. Just to feel they’re keeping in touch with you and they know you’re waiting.” (P9)  “Now I can put a request in [to my GP], that I can think about, as an email. And somebody will get back to me, as an email, and then I can think about it a bit, and then I can get back to them. So, in a sense, that builds in the pauses. That is more helpful for me.” (P13)  “But I see nothing wrong with it not being an aim for each GP practice of a particular size and above to have a person who understands what being autism aware means. And what the implications might be.” (P17) |
| 4 | **Sensory-friendly practice** | “Some of them [health professionals] use that disinfectant wipe or whatever it is to put on your arms when they do blood tests. That can put me in one [a ‘meltdown’] because that’s too strong and that’s right by me.” (P6)  “One person said [bring] earplugs and eye mask [to the hospital]. And I thought I won’t need those. But I really should have taken them with me. Because there was so much noise all the time and people talking and lights on. And awful smells of the polish and the food they were cooking for every meal, especially when you feel you don’t want to eat it anyway.” (P10)  “Have you come across the Spoon Theory? That you have so many spoons of energy per day, and that each thing that you have to do uses a spoon. Basically, all those spoons are gone in coping with the lighting, getting lost, being worried about getting lost, being worried about doing the wrong thing, all that.” (P19) |
| 5 | **Empathy and reassurance** | “So I think it's really important for each person with autism just to be aware of what their particular needs are in any given situation. And for the person on the other end just to be aware of that and make allowances.” (P1)  “I had one wonderful GP once. I used to go with a piece of paper in my hand, with everything that I was worried about written on it. And he’d just take it from my hand, read it, and answer the questions one by one. That was great.” (P7)  "If I say, I'm autistic and I also have anxiety, they say okay, anytime that you feel you want to stop. […] The ones that are genuine, they will, whatever they're doing, they'll say, is this, okay? Is that okay? I'm just going to hold your hand, they will explain to you, I'm just going to read your pulse here." (P11) |
| 6 | **Communication and processing style** | “I find it easier that you can answer a direct question… almost yes or no, or does this hurt? Does that hurt? Or do you find it here? Rather than me trying to explain what hurts. Sometimes, short, bullet-type questions for someone like me, which I think they want to avoid usually.” (P4)  “I think that’s really important because so often, they just rush on from one thing to the next. And I've completely lost them by the time they’ve finished because my brain can’t keep up with that speed.” (P9)  “I said to her [physiotherapist], if I can see something, I can make sense of it. And she said give me your phone I’ll just transfer the X-ray. And do you know what that was so helpful because […] I could understand why I get pain in a certain place and why certain movements would trigger it.” (P15) |
| 7 | **Continuity of care** | “This last doctor [I saw in A&E] was absolutely amazing and just hit the nail on the head really with regards to what I needed in terms of information, in terms of having a named person, I think that's important. Seeing your care through to the end and giving you some idea about how long you'd have to wait in each department and just that reassurance.” (P1)  “I told the ambulance crew that I realise I needed treatment for autism and they did contact the hospital before we got there and they did arrange for me to be put in an end-bed in as quiet a place as possible. So, the hospital knew that I was using these aids for self-survival, so they were happy with that.” (P2)  “[At the dentist] you have to fill in a form about any diagnosis you have. So one day, I just added Asperger’s syndrome to it. And as I walked into the hygienist, she said now, what can I do that’s helpful? Amazing. So I said well, turn the music down. So she turned it, actually, off. And now, she knows I’m coming in, she just turns it off.” (P7) |
